# Supplementary figures and images for: Intrinsic connectivity reveals functionally distinct cortico-hippocampal networks in the human brain
Source: PLoS Biol. 2021 Jun 2;19(6):e3001275. doi: 10.1371/journal.pbio.3001275 (PMC8202937; doi:10.1371/journal.pbio.3001275)

Group 1

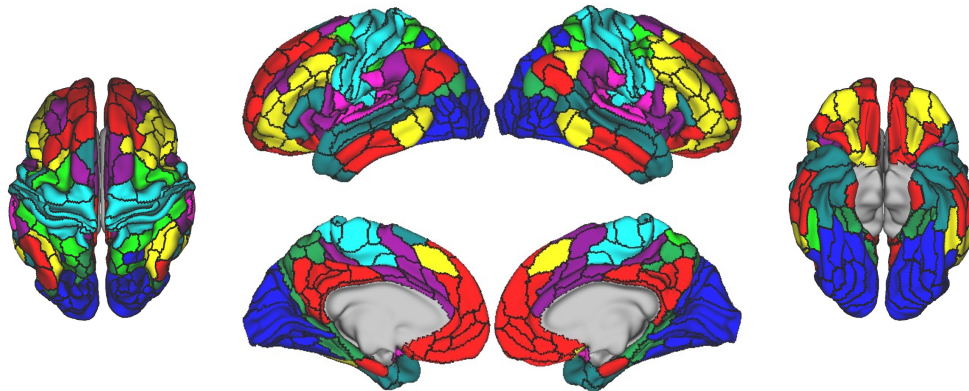

Group 2

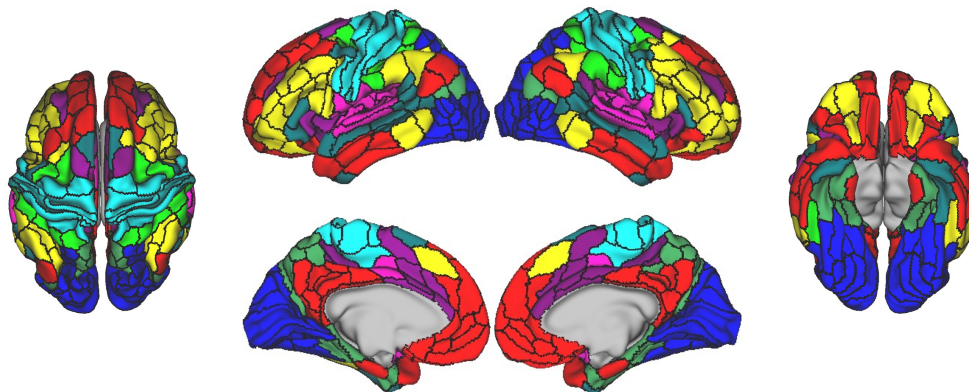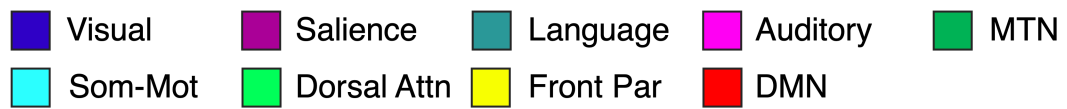

Supplement: S1 Fig — Attn, attention; DMN, default mode network; Front Par, frontoparietal; MTN, medial temporal network; Som-Mot, somatomotor. (PDF) [file pbio.3001275.s001.pdf]

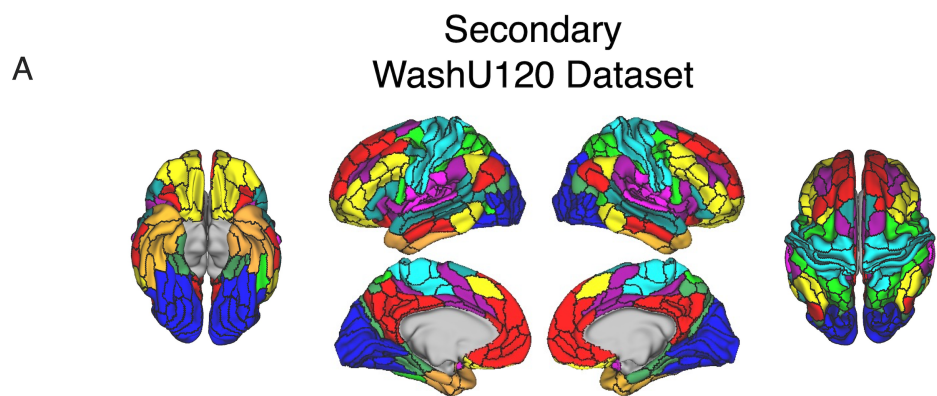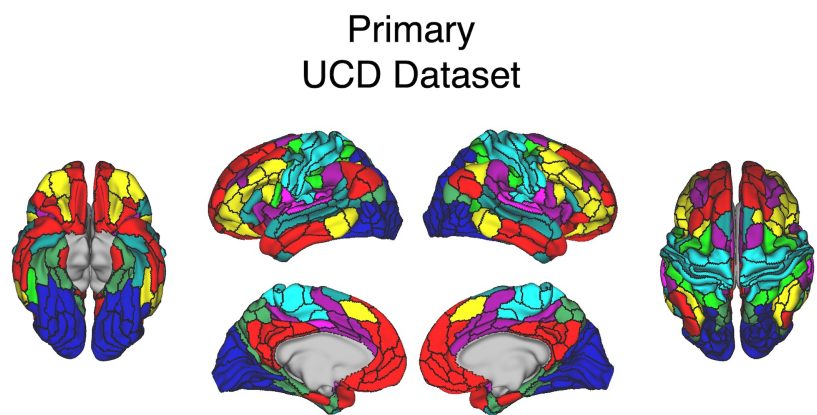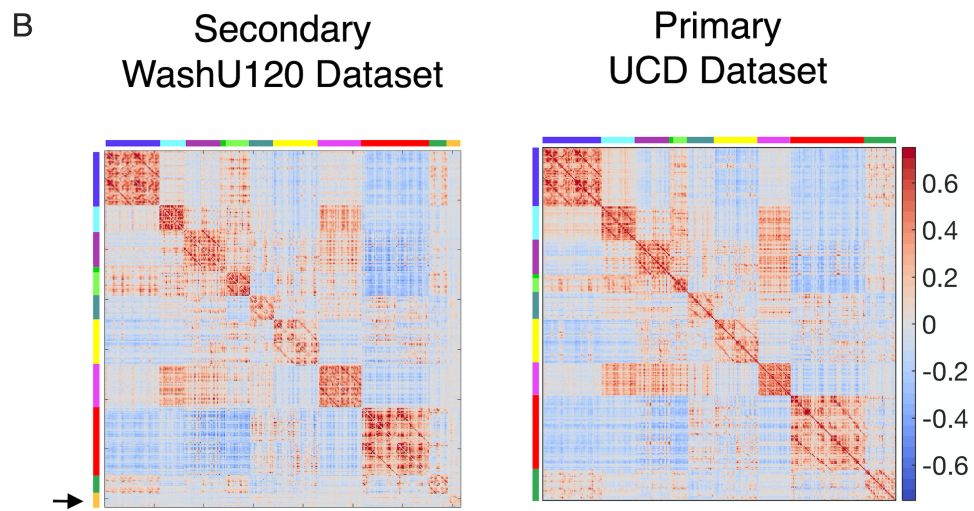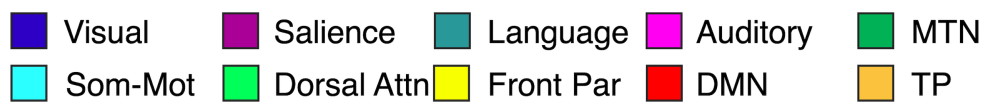

Supplement: S2 Fig — (A) Inflated cortical surface, colored according to community membership for the secondary WashU120 dataset (top) and primary UCD dataset (bottom). (B) Connectivity matrix reordered by community to demonstrate the community structure of the group-averaged brain from the Secondary WashU120 dataset (left) and primary UCD dataset (right). Colors along the axis demonstrate which rows/columns belong to a given community. Color bar represents Fisher Z-transformed correlation values. Arrow highlights the temporal polar network in the WashU120 dataset that has overall low (near 0) whole-brain FC. Attn, attention; DMN, default mode network; FC, functional connectivity; Front Par, frontoparietal; MTN, medial temporal network; Som-Mot, somatomotor. TP, Temporal polar; UCD, University of California, Davis. Data can be found at https://github.com/ajbarn/hippo_nets. (PDF) [file pbio.3001275.s002.pdf]

A.

Primary  
UCD Dataset

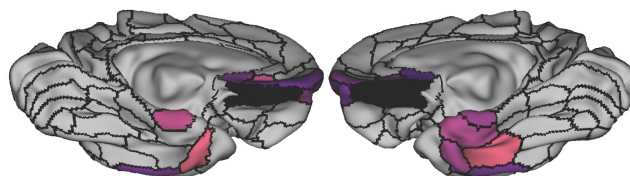

Secondary  
WashU120 Dataset

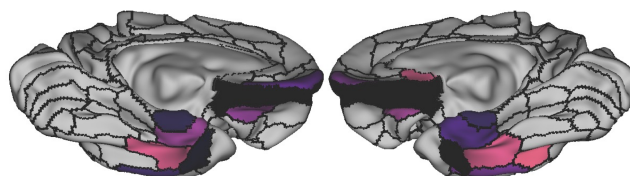

14.8 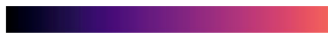 30  
tSNR

B.

Secondary > Primary

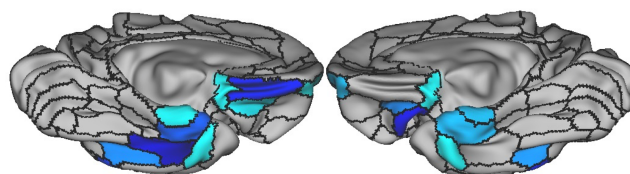

-2 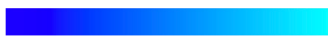 -5  
t stat

Supplement: S3 Fig — A. The tSNR on the ventral surface of an inflated brain for regions with low tSNR (tSNR < 30) for the primary (UCD) and secondary (WashU120) datasets. B. The t-statistics for the difference in tSNR between the primary and secondary datasets. Regions in blue are those that showed significantly lower tSNR in the secondary dataset compared to the primary dataset. tSNR, temporal signal-to-noise ratio; UCD, University of California, Davis. Data can be found at https://github.com/ajbarn/hippo_nets. (PDF) [file pbio.3001275.s003.pdf]

X-axis = removed network

Y-axis = path length between DMN and target following removal of network

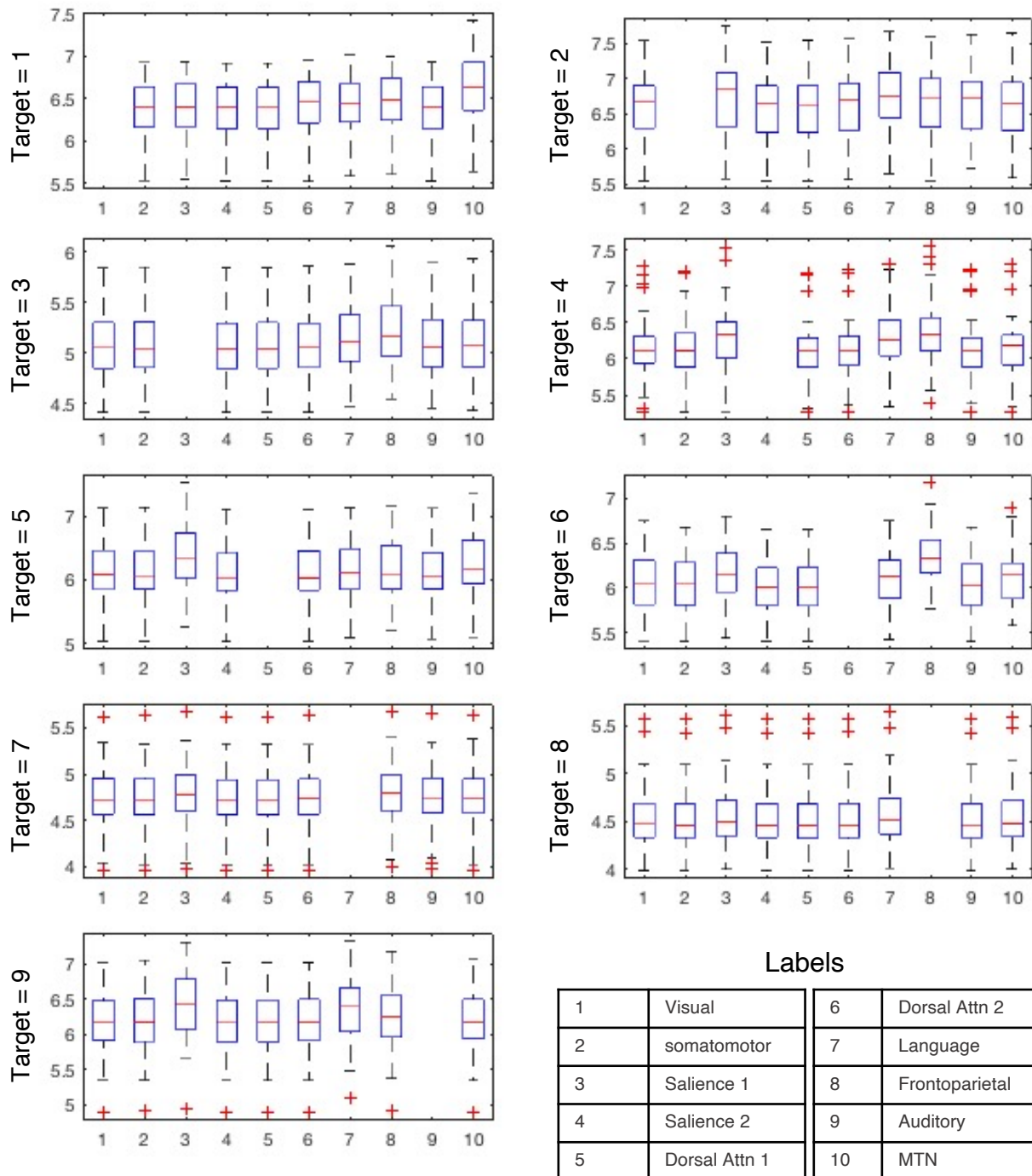

Supplement: S4 Fig — Data can be found at https://github.com/ajbarn/hippo_nets. DMN, default mode network. (PDF) [file pbio.3001275.s004.pdf]

### Target: MTN

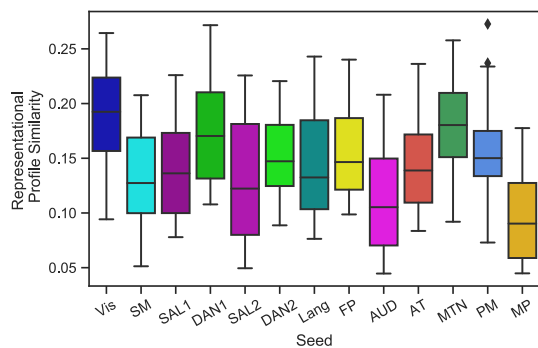

### Target: AT

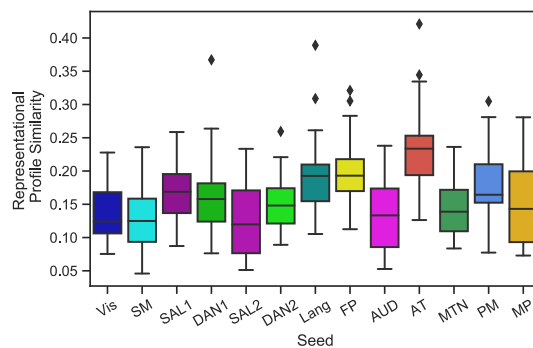

### Target: PM

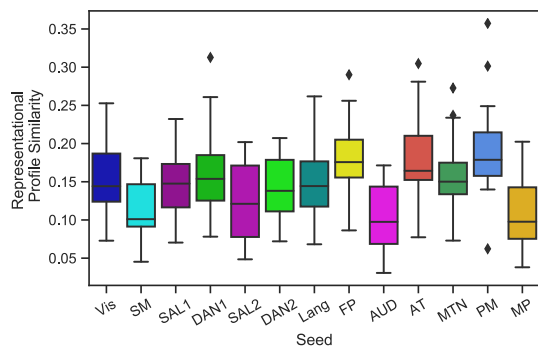

### Target: MP

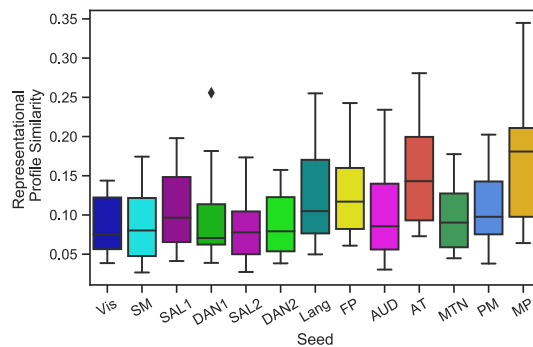

Supplement: S6 Fig — AUD, auditory; AT, anterior temporal; DAN1, dorsal attention network 1; DAN2, dorsal attention network 2; FP, frontoparietal; Lang, language; MP, medial prefrontal; MTN, medial temporal network; PM, posterior medial; SAL1, salience 1; SAL2, salience 2; SM, somatomotor; VIS, visual. Data can be found at https://github.com/ajbarn/hippo_nets. (PDF) [file pbio.3001275.s006.pdf]
