# Supplementary material for: Intrinsic connectivity reveals functionally distinct cortico-hippocampal networks in the human brain
Source: PLoS Biol. 2021 Jun 2;19(6):e3001275. doi: 10.1371/journal.pbio.3001275 (PMC8202937; doi:10.1371/journal.pbio.3001275)
Supplement: S5 Fig — Data can be found at https://github.com/ajbarn/hippo_nets. (PDF) [file pbio.3001275.s005.pdf]

X-axis = removed network
Y-axis = path length between hippocampus and target following removal of network

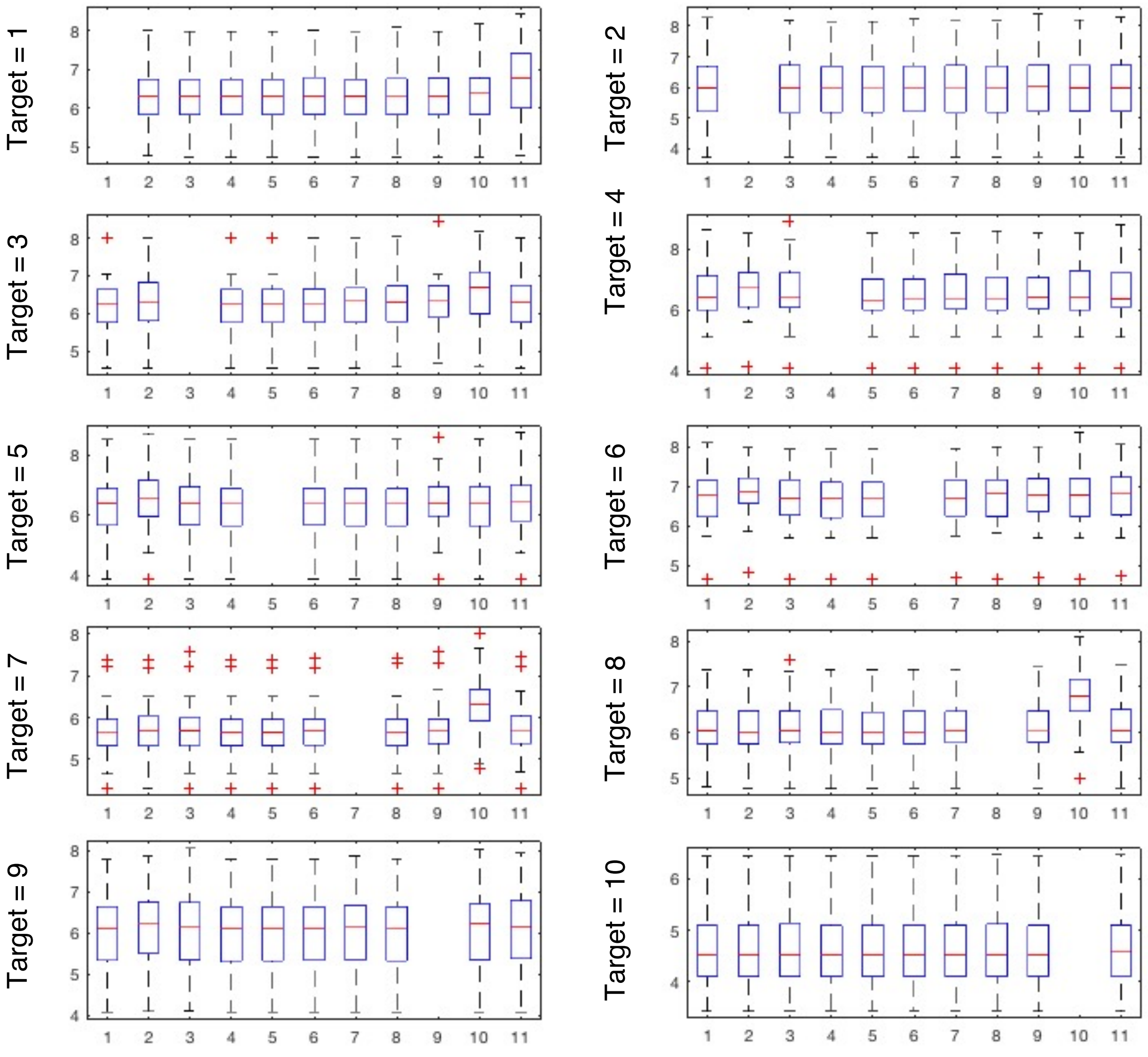

Labels

1	Visual	6	Dorsal Attn 2	11	MTN
2	somatomotor	7	Language		
3	Salience 1	8	Frontoparietal		
4	Salience 2	9	Auditory		
5	Dorsal Attn 1	10	DMN		
