# Supplementary material for: Intrinsic connectivity reveals functionally distinct cortico-hippocampal networks in the human brain
Source: PLoS Biol. 2021 Jun 2;19(6):e3001275. doi: 10.1371/journal.pbio.3001275 (PMC8202937; doi:10.1371/journal.pbio.3001275)
Supplement: S1 Table — FC represented as the Fisher z-transformed correlation between the functional time series between 2 regions. FC, functional connectivity. (DOCX) [file pbio.3001275.s007.docx]

S1 Table. Mean of the average functional connectivity between the hippocampus and the regions within each cortical network across subjects with standard deviation in parenthesis. Functional connectivity represented as the Fisher z-transformed correlation between the functional timeseries between two regions.

| Network | Right Posterior | Left Posterior | Right Anterior | Left Anterior |
| --- | --- | --- | --- | --- |
| Visual | -0.005 (.07) | -.027 (.07) | -.013 (.07) | -.031 (.08) |
| Somatomotor | .038 (.07) | .022 (.07) | **.063 (.08)** | **.052 (.09)** |
| Cingulo-Opercular 1 | -.021 (.06) | -.020 (.06) | -.089 (.07) | -.069 (.08) |
| Cingulo-Opercular 2 | -.091 (.1) | -.098 (.11) | -.084 (.12) | -.081 (.1) |
| DAN 1 | -.012 (.11) | -.042 (.11) | -.097 (.14) | -.084 (.14) |
| DAN 2 | -.053 (.06) | -.066 (.06) | -.073 (.07) | -.081 (.07) |
| Language | -.016 (.04) | -.004 (.05) | **.041 (.05)** | **.041 (.05)** |
| Frontoparietal | -.043 (.04) | -.032 (.04) | -.034 (.05) | -.034 (.04) |
| Auditory | .03 (.07) | .025 (.08) | .020 (.08) | .032 (.08) |
| DMN | **.082 (.05)** | **.092 (.05)** | **.111 (.06)** | **.118 (.06)** |
| MTL | **.056 (.04)** | **.038 (.04)** | **.042 (.05)** | **.028 (.04)** |

DAN, Dorsal Attention Network; DMN default mode network; MTL, medial temporal lobe network; Bold values are significant at p<.05, Bonferroni-corrected.
