# Supplementary material for: Intrinsic connectivity reveals functionally distinct cortico-hippocampal networks in the human brain
Source: PLoS Biol. 2021 Jun 2;19(6):e3001275. doi: 10.1371/journal.pbio.3001275 (PMC8202937; doi:10.1371/journal.pbio.3001275)
Supplement: S2 Table — (DOCX) [file pbio.3001275.s008.docx]

S2 Table. Correlation values of spatial overlap between the cortico-hippocampal networks and meta-analytic maps for cognitive terms.

| Cognitive Term | MTN | PM | AT | MP |
| --- | --- | --- | --- | --- |
| episodic | 0.2009 | 0.1455 | 0.0634 | 0.0366 |
| navigation | 0.1499 | -0.0038 | -0.0462 | -0.0413 |
| confidence | 0.1479 | 0.0668 | 0.0169 | 0.0161 |
| retrieval | 0.144 | 0.0282 | 0.0576 | 0.0078 |
| semantic memory | 0.1425 | 0.0719 | 0.0615 | 0.0422 |
| autobiographical | 0.1411 | 0.1427 | 0.1231 | 0.096 |
| recollection | 0.1375 | 0.1062 | 0.0727 | 0.0212 |
| construction | 0.1289 | 0.067 | 0.0763 | 0.0398 |
| encoding | 0.1268 | 0.0058 | -0.011 | -0.0354 |
| scene | 0.119 | 0.0076 | 0.0323 | -0.0046 |
| past | 0.1175 | 0.1026 | 0.0863 | 0.068 |
| recall | 0.1133 | 0.0732 | 0.0574 | 0.0201 |
| remembering | 0.1029 | 0.0915 | 0.0571 | 0.0379 |
| details | 0.1013 | 0.0892 | 0.0356 | 0.0409 |
| recognition | 0.0991 | 0.0526 | 0.0177 | -0.0298 |
| mental states | 0.0377 | 0.0659 | 0.2203 | 0.047 |
| theory mind | 0.0401 | 0.0785 | 0.2154 | 0.0631 |
| social | 0.0394 | 0.051 | 0.188 | 0.0379 |
| intentions | 0.0263 | 0.0193 | 0.1696 | 0.0232 |
| referential | 0.0616 | 0.1285 | 0.1479 | 0.0995 |
| person | 0.0529 | 0.0511 | 0.1473 | 0.0688 |
| moral | 0.0246 | 0.0729 | 0.147 | 0.0906 |
| beliefs | 0.0246 | 0.0514 | 0.1454 | 0.0521 |
| self referential | 0.061 | 0.1315 | 0.1454 | 0.0961 |
| social interaction | 0.0028 | -0.0078 | 0.1382 | 0.0379 |
| mentalizing | 0.0555 | 0.0788 | 0.1381 | 0.0544 |
| intention | 0.0382 | 0.0662 | 0.1242 | 0.0529 |
| traits | -0.0141 | 0.0039 | 0.1224 | 0.1244 |
| judgments | 0.0527 | 0.071 | 0.1109 | 0.0274 |
| memory retrieval | 0.1223 | 0.1323 | 0.0887 | 0.0514 |
| self | 0.061 | 0.1131 | 0.1404 | 0.1266 |
| mental | 0.0732 | 0.1061 | 0.1219 | -0.0033 |
| recognition memory | 0.0735 | 0.1009 | 0.0253 | -0.0232 |
| mental state | 0.081 | 0.083 | 0.1306 | 0.04 |
| personal | 0.0937 | 0.0755 | 0.1057 | 0.1002 |
| value | 0.0068 | 0.0321 | 0.0515 | 0.1706 |
| fear | -0.0059 | -0.0284 | 0.0041 | 0.1591 |
| emotion | 0.0027 | -0.0032 | 0.0987 | 0.156 |
| trait | -0.0109 | 0.0306 | 0.1134 | 0.1422 |
| valence | 0.0124 | 0.0052 | 0.0861 | 0.1415 |
| preferences | -0.0208 | 0.0059 | 0.0209 | 0.13 |
| social | 0.0394 | 0.0378 | 0.188 | 0.1298 |
| regulation | -0.0061 | 0.0044 | 0.0763 | 0.1284 |
| decision making | -0.0433 | 0.0404 | 0.0035 | 0.1272 |
| reward | -0.0322 | -0.0081 | -0.0101 | 0.1272 |
| personality | -0.0062 | 0.0254 | 0.1044 | 0.1267 |
| threat | 0.0107 | -0.023 | -0.0017 | 0.1234 |
| anxiety | 0.0179 | 0.0065 | 0.0271 | 0.1231 |
| arousal | -0.0055 | -0.0114 | 0.0389 | 0.1172 |
