# Supplementary material for: Intrinsic connectivity reveals functionally distinct cortico-hippocampal networks in the human brain
Source: PLoS Biol. 2021 Jun 2;19(6):e3001275. doi: 10.1371/journal.pbio.3001275 (PMC8202937; doi:10.1371/journal.pbio.3001275)
Supplement: S3 Table — AT, anterior temporal; MP, medial prefrontal; MTN, medial temporal network; PM, posterior medial. (DOCX) [file pbio.3001275.s009.docx]

S3 Table. Regions of interest in the HCP-MMP atlas 1.0 that are found in the Medial Temporal network (MTN), Anterior temporal (AT) subnetwork, Posterior medial (PM) subnetwork, and Medial Prefrontal (MP) Subnetwork, with their community label provided.

| Region of Interest | Community | Name |
| --- | --- | --- |
| L_POS2_ROI | MTN | Parieto-Occipital_Sulcus_Area_2 |
| L_PCV_ROI | MTN | PreCuneus_Visual_Area |
| L_7Pm_ROI | MTN | Medial Area 7P |
| L_7Am_ROI | MTN | Medial Area 7A |
| L_7Pl_ROI | MTN | Lateral Area 7P |
| L_PreS_ROI | MTN | Presubiculum |
| L_ProS_ROI | MTN | ProStriate Area |
| L_PeEc_ROI | MTN | Perirhinal Ectorhinal Cortex |
| L_PHA1_ROI | MTN | Parahippocampal Area 1 |
| L_PHA3_ROI | MTN | Parahippocampal Area 3 |
| L_TPOJ3_ROI | MTN | Area  TemporoParietoOccipital Junction 3 |
| L_DVT_ROI | MTN | Dorsal Transitional  Visual Area |
| L_PGp_ROI | MTN | Area PGp |
| L_PHA2_ROI | MTN | Parahippocampal Area 2 |
| R_POS2_ROI | MTN | Parieto-Occipital Sulcus Area 2 |
| R_PCV_ROI | MTN | PreCuneus Visual  Area |
| R_7Pm_ROI | MTN | Medial Area 7P |
| R_POS1_ROI | MTN | Parieto-Occipital Sulcus Area 1 |
| R_7Am_ROI | MTN | Medial Area 7A |
| R_7PL_ROI | MTN | Lateral Area 7P |
| R_PreS_ROI | MTN | Presubiculum |
| R_ProS_ROI | MTN | ProStriate Area |
| R_PeEc_ROI | MTN | Perirhinal Ectorhinal Cortex |
| R_PHA1_ROI | MTN | Parahippocampal Area 1 |
| R_PHA3_ROI | MTN | Parahippocampal Area 3 |
| R_TF_ROI | MTN | Area TF |
| R_TPOJ3_ROI | MTN | Area  TemporoParietoOccipital Junction 3 |
| R_PGp_ROI | MTN | Area PGp |
| R_IP0_ROI | MTN | Area IntraParietal 0 |
| R_PHA2_ROI | MTN | Parahippocampal Area 2 |
| L_23d_ROI | AT | Area 23d |
| L_d32_ROI | AT | Area dorsal 32 |
| L_8Av_ROI | AT | Area 8Av |
| L_9m_ROI | AT | Area 9 Middle |
| L_8BL_ROI | AT | Area 8B Lateral |
| L_9p_ROI | AT | Area 9 Posterior |
| L_10d_ROI | AT | Area 10d |
| L_9a_ROI | AT | Area 9 anterior |
| L_a10p_ROI | AT | Area anterior 10p |
| L_47s_ROI | AT | Area 47s |
| L_TGd_ROI | AT | Area TG dorsal |
| L_TE1a_ROI | AT | Area TE1 anterior |
| L_TE2a_ROI | AT | Area TE2 anterior |
| L_PFm_ROI | AT | Area PFm Complex |
| L_PGi_ROI | AT | Area PGi |
| L_STSva_ROI | AT | Area STSv anterior |
| L_TE1m_ROI | AT | Area TE1 Middle |
| R_d32_ROI | AT | Area dorsal 32 |
| R_9m_ROI | AT | Area 9 Middle |
| R_8BL_ROI | AT | Area 8B Lateral |
| R_9p_ROI | AT | Area 9 Posterior |
| R_10d_ROI | AT | Area 10d |
| R_47l_ROI | AT | Area 47 lateral |
| R_9a_ROI | AT | Area 9 anterior |
| R_47s_ROI | AT | Area 47s |
| R_TGd_ROI | AT | Area TG dorsal |
| R_TE1a_ROI | AT | Area TE1 anterior |
| R_TE2a_ROI | AT | Area TE2 anterior |
| R_STSva_ROI | AT | Area STSv anterior |
| R_TE1m_ROI | AT | Area TE1 Middle |
| L_RSC_ROI | PM | RetroSplenial Complex |
| L_7m_ROI | PM | Area 7m |
| L_POS1_ROI | PM | Parieto-Occipital  Sulcus Area 1 |
| L_v23ab_ROI | PM | Area ventral 23 a+b |
| L_d23ab_ROI | PM | Area dorsal 23 a+b |
| L_31pv_ROI | PM | Area 31p ventral |
| L_8Ad_ROI | PM | Area 8Ad |
| L_s6-8_ROI | PM | Superior 6-8  Transitional Area |
| L_PGs_ROI | PM | Area PGs |
| L_31pd_ROI | PM | Area 31pd |
| L_31a_ROI | PM | Area 31a |
| L_p10p_ROI | PM | Area posterior 10p |
| R_RSC_ROI | PM | RetroSplenial Complex |
| R_7m_ROI | PM | Area 7m |
| R_23d_ROI | PM | Area 23d |
| R_v23ab_ROI | PM | Area ventral 23 a+b |
| R_d23ab_ROI | PM | Area dorsal 23 a+b |
| R_31pv_ROI | PM | Area 31p ventral |
| R_8Ad_ROI | PM | Area 8Ad |
| R_PGi_ROI | PM | Area PGi |
| R_PGs_ROI | PM | Area PGs |
| R_31pd_ROI | PM | Area 31pd |
| R_31a_ROI | PM | Area 31a |
| R_p10p_ROI | PM | Area posterior 10p |
| L_a24_ROI | MP | Area a24 |
| L_p32_ROI | MP | Area p32 |
| L_10r_ROI | MP | Area 10r |
| L_47m_ROI | MP | Area 47m |
| L_10v_ROI | MP | Area 10v |
| L_10pp_ROI | MP | Polar 10p |
| L_OFC_ROI | MP | Orbital Frontal Complex |
| L_EC_ROI | MP | Entorhinal Cortex |
| L_25_ROI | MP | Area 25 |
| L_s32_ROI | MP | Area s32 |
| L_pOFC_ROI | MP | posterior OFC Complex |
| R_a24_ROI | MP | Area a24 |
| R_p32_ROI | MP | Area p32 |
| R_10r_ROI | MP | Area 10r |
| R_47m_ROI | MP | Area 47m |
| R_10v_ROI | MP | Area 10v |
| R_10pp_ROI | MP | Polar 10p |
| R_OFC_ROI | MP | Orbital Frontal Complex |
| R_EC_ROI | MP | Entorhinal Cortex |
| R_25_ROI | MP | Area 25 |
| R_s32_ROI | MP | Area s32 |
| R_pOFC_ROI | MP | posterior OFC Complex |
