# Supplementary material for: Intrinsic connectivity reveals functionally distinct cortico-hippocampal networks in the human brain
Source: PLoS Biol. 2021 Jun 2;19(6):e3001275. doi: 10.1371/journal.pbio.3001275 (PMC8202937; doi:10.1371/journal.pbio.3001275)
Supplement: S1 Text — (DOCX) [file pbio.3001275.s010.docx]

Supplemental Methods

*Pattern similarity analysis*

Multivoxel pattern similarity analyses were performed on the fMRI data from the decision task runs. The analyses were conducted on unsmoothed functional images in the native space and the EPI timeseries underwent motion correction and high-pass filtering (0.01 Hz) in FMRIB’s Software Library (FSL; https://fsl.fmrib.ox.ac.uk/fsl/fslwiki).

For each trial, a single beta image was estimated by single trial models, based on the approach called Least Squares Single (LSS), for event-related blood oxygenation level-dependent (BOLD) signal change, controlling for signal change due to all other trials and motion artifact, using ordinary least squares regression, resulting in 128 single-trial beta images (1,2). Parameter estimates for each trial were computed using a general linear model, with the first regressor as a stick function placed at the onset of each trial and a second regressor containing all the other trials.

Single-trial beta images from run 2 were coregistered with single-trial beta images from run 1 using FSL’s FLIRT linear registration software (6 degrees of freedom). Coregistered single-trial beta images with atypically high mean absolute z-score (based on the distribution of beta estimates for each grey matter voxel across all trials) were excluded from further analysis. Based on a mean absolute z threshold of 1.5, between 0 and 10 trials were excluded per subject with a median of 4. Beta images went through a second visual inspection to make sure all the deviant trials were excluded. This noise trial exclusion procedure is adopted from the previous pattern similarity studies in the lab (3).

The representational similarity analyses were then conducted using the RSA toolbox by Nili et al. (4). For each region of interest, all trial patterns were correlated with each other using Pearson's r resulting in a 128 * 128 pattern similarity matrix for each ROI. The 128 * 128 pattern similarity matrix for each ROI was then filtered to exclude pairwise combinations that consisted of a trial with a bad quality beta. Then, the filtered 128 * 128 pattern similarity matrices for each ROI were vectorized and correlated for each pair of ROIs using Spearman’s correlation to assess the similarity of the representational profiles.

Supplemental References

1. Mumford JA, Turner BO, Ashby FG, Poldrack RA. Deconvolving BOLD activation in event-related designs for multivoxel pattern classification analyses. Neuroimage [Internet]. 2012;59(3):2636–43. Available from: http://dx.doi.org/10.1016/j.neuroimage.2011.08.076

2. Mumford JA, Davis T, Poldrack RA. The impact of study design on pattern estimation for single-trial multivariate pattern analysis. Neuroimage [Internet]. 2014;103:130–8. Available from: http://dx.doi.org/10.1016/j.neuroimage.2014.09.026

3. Libby LA, Reagh ZM, Bouffard NR, Ragland JD, Ranganath C. The Hippocampus Generalizes across Memories that Share Item and Context Information. J Cogn Neurosci [Internet]. 2019 Jan;31(1):24–35. Available from: http://dx.doi.org/10.1162/jocn_a_00409%5Cnhttp://www.mitpressjournals.org/doi/abs/10.1162/jocn_a_00409

4. Nili H, Wingfield C, Walther A, Su L, Marslen-Wilson W, Kriegeskorte N. A Toolbox for Representational Similarity Analysis. Prlic A, editor. PLoS Comput Biol [Internet]. 2014 Apr 17;10(4):e1003553. Available from: https://dx.plos.org/10.1371/journal.pcbi.1003553
