# Supplementary material for: Intrinsic connectivity reveals functionally distinct cortico-hippocampal networks in the human brain
Source: PLoS Biol. 2021 Jun 2;19(6):e3001275. doi: 10.1371/journal.pbio.3001275 (PMC8202937; doi:10.1371/journal.pbio.3001275)
Supplement: Attachment — Submitted filename: Response_to_reviewers_Revision.docx [file pbio.3001275.s011.docx]

REVIEWS:

**Reviewer #1:** In this paper, Barnett et al. seek to clarify the relationship between medial temporal lobes (MTL) and default mode (DMN) subnetworks. Past work has identified multiple subnetworks of the MTL and hippocampus that underlie memory-guided behavior; somewhat similarly, data-driven analyses have parcellated the DMN. Seeking to unify these approaches, the current studies characterize cortico-hippocampal networks and their relationship to episodic memory. They find that portions of the DMN and medial temporal lobes segregate from the rest of the DMN to form a medial temporal network (MTN). The MTN then seems to be responsible for transferring information from the visual network to the DMN and hippocampus. The DMN was further subdivided into 3 smaller subnetworks, which partly vary in their connectivity with the hippocampus and their functionality.

Overall, I found the paper to be solid and interesting to read. These networks are important to understand, and the relationship to function presented at the end was clever. However, one of the first findings described in the paper is that a network partitioning (using a community detection algorithm) pulls out the DMN, but only in part. A subset of traditionally DMN regions (parahippocampal cortex, retrosplenial cortex, and perirhinal cortex) instead grouped with some MTL and parietal regions. Further analyses in the paper really hinge on this relatively novel subdivision, so I think it deserves more scrutiny. Would the same algorithms applied to different (perhaps publicly available) datasets produce the same divisions? On the 40 subjects used here, would other methods of extracting the DMN produce more traditional results? Similarly, what would happen if the analyses associated with Figure 3 were repeated, but combining the DMN and MTN? Or using a different definition of

the DMN? Essentially, I think that the notion of the division between the DMN and the MTN needs to be more fully explored, before the subdivisions of the DMN are tackled. I think the major impact of the paper lies here, and it will bring it to a level above the (many) DMN parcellation papers that already exist.

**We have strived to increase our confidence in our initial community partition in two ways. First, we performed a split half analysis in which we split the original dataset into two equal halves with comparable demographic details and re-ran the community detection. We observed high similarity between the groups and also replicated the distinction between the MTN and DMN. Second, we downloaded a publicly available dataset (WashU dataset available at** [**https://openneuro.org/datasets/ds000243/versions/00001**](https://openneuro.org/datasets/ds000243/versions/00001)**) of which we had 69 usable participants following preprocessing and data quality inspection. Using this additional dataset, we re-ran the community detection following identical preprocessing procedures. For this, we again found high labelling consistency with our original sample and also observed a distinction between the MTN and DMN. These replications gave us considerable confidence in the distinction between the MTN and DMN.**

**As we note in the revised manuscript, the only major difference in network parcellations across the two datasets was that, in the secondary dataset, anterior temporal lobe regions differentiated into a single community that was disconnected from all of the other networks. We found that these regions had low temporal signal to noise in the secondary dataset. Our acquisition sequence was optimized for data acquisition in the medial temporal lobes, and as such, we can be confident in our parcellation of the anterior temporal subnetwork in the UC Davis dataset.**

**These results are described on page 6 and 7 and are shown below:**

To provide internal validation of our partition, we split our dataset into two equal sized subsamples and repeated the community detection procedures. We observed very high consistency across the two split-halves, with an adjusted z-Rand index of z = 172, p <.0001 and 95% of regions were given the same label in both halves comparable to the split-half analysis performed by Ji et al (43) (split half results shown in S1 Figure). Importantly, we observed the same expression of resting networks, including the DMN and MTN.

S1 Figure Caption

Inflated cortical surface, colored according to community membership for community detection analyses performed on split halves of the sample. Attn, attention; DMN, Default mode network; Front Par, Frontoparietal; MTN, Medial temporal network; Som-Mot, Somatomotor.

        To provide external validation of our partition, we acquired an openly available resting state fMRI dataset from OpenNeuro.org acquired on a 3T scanner (https://openneuro.org/datasets/ds000243/versions/00001). We used 69 participants from this dataset following preprocessing and data quality checks. The total scan length, per subject, was 12.8 minutes on average and methodological details have been reported in previous publications (52, 53). Community detection was, again, repeated on this secondary dataset following identical preprocessing procedures and we observed comparable delineations of these canonical networks (shown in S2 Figure A). Between the primary and secondary datasets, we observed strong partition consistency with an adjusted z-Rand index of z = 150, p <.0001 and 93% of regions were given the same label in both datasets. The only major difference in network parcellations across the two datasets was that, in the secondary dataset, anterior temporal lobe regions differentiated into a single community that was disconnected from all of the other networks. We observed that these regions had low functional connectivity (S2 Figure B) and low temporal signal to noise (tSNR) in the secondary dataset (see S3 Figure for comparison of tSNR between datasets). Accordingly, there is good reason to believe that the isolated network of anterior temporal regions in the secondary dataset is related to poor data quality in the anterior temporal lobes. Given the results of the split half analysis reported above, and the fact that our acquisition sequence showed data quality in the medial and anterior temporal lobes, we can be confident in the identification of the anterior temporal subnetwork in the UC Davis dataset.

S2 Figure Caption

A. Inflated cortical surface, colored according to community membership for the secondary WashU120 dataset (top) and primary UCD dataset (bottom). B. Connectivity matrix reordered by community to demonstrate the community structure of the group-averaged brain from the Secondary WashU120 dataset (left) and primary UCD dataset (right). Colors along the axis demonstrate which rows/columns belong to a given community. Color bar represents Fisher Z-transformed correlation values. Arrow highlights the temporal polar network in the WashU120 dataset that has overall low (near zero) whole-brain functional connectivity. Attn, attention; DMN, Default mode network; Front Par, Frontoparietal; MTN, Medial temporal network; Som-Mot, Somatomotor. TP, Temporal polar

S3 Figure Caption

A. The temporal signal to noise ratio (tSNR) on the ventral surface of an inflated brain for regions with low tSNR (tSNR<30) for the primary (University of California, Davis; UCD) and secondary (WashU120) datasets. B. The t-statistics for the difference in tSNR between the primary and secondary datasets. Regions in blue are those that showed significantly lower tSNR in the secondary dataset compared to the primary dataset.

**The methods for these analyses have also been added to the methods section and are integrated with the analyses steps of the manuscript.**

Minor comments

There are so many abbreviations, I think it would help the reader enormously to cut back. DMN and MTL are fine, but some combination of PHC, PRC, MTN, PM, AT, MP should just be spelled out.

**We thank the reviewer for this suggestion and have removed the acronyms for PHC and PRC from the manuscript.**

I found the analysis and accompanying description for Figure 2 very satisfying and interesting.

**Thank you!**

Line 193—lead should be led

**We have updated the manuscript to fix this error.**

Line 442—on should be of

**We have updated the manuscript to fix this error**

**Reviewer #2:** Barnett and colleagues conducted a thorough examination of the resting state connectivity structure of human cortico-hippocampal networks. Using a sample of 40 participants with 25-minutes of resting state functional connectivity, alongside an array graph theory-based analyses, the authors report a series of interesting results regarding the organization of large-scale brain networks and their relationship to the hippocampus:

(1)	A replication of previous studies highlighting a distinct medial temporal network (MTN), separate from the default mode network (DMN)

(2)	The MTN's role in mediating communication between the DMN and visual network (VN)

(3)	Demonstrating the DMN is composed of separable subnetworks, with new insights regarding how these different subnetworks may mediate communication within and outside of the DMN

(4)	The relationship between the hippocampal long-axis and these separable DMN subsystems

The authors also included additional task-data and existing meta-analytic tools (i.e., Neurosynth) to extend these findings to how these regions may represent psychologically-meaningful information content.

Overall, I found this manuscript very relevant to current theories of hippocampal-whole brain macro-level organization. The analyses were thorough. The writing was clear. It was a pleasure to read.

I have a few points that I hope will further benefit the quality of this manuscript and its conclusions.

Major comments:

(1)	Can spatial proximity explain the role of the MTN in mediating crosstalk between DMN and VN?

The analysis examining the role of the MTN in mediating communication between the DMN/hippocampus and VN is particularly interesting. I appreciate the control analyses the authors have conducted, but I wonder whether this effect might be explained by spatial proximity of the MTN to both the DMN/hippocampus and VN. In other words, is the MTN the most spatially proximal network to both the DMN/hippocampus and VN simultaneously? Could spatial proximity/euclidean distance alone explain the observed path length effect? Or, is it something more about the underlying anatomy/functional coupling of these specific regions?

Given that FC tends to have sharp boundaries, it's unlikely that spatial proximity alone could account for this effect. But including a control analysis that examines this possibility can further clarify why the MTN should mediate communication between the DMN/HPC and VN.

First, the authors can calculate the spatial proximity of each ROI to each other, and then average within network. [Note: the authors already did this as a control for the RSA analysis]. Is the MTN the network that is simultaneously closest to both DMN and VN/HPC and VN? If so, a potential control analysis could be to redo the 1000 resample control where 30 ROIs were randomly removed, but add an additional constraint that the 30 need to have at least the same (or some relatively comparable level) spatial proximity to both the DMN and VN as the MTN. This way, in addition to number of nodes in a network, the authors can control for the euclidean distance between the ROIs and the two target networks they purportedly mediate.

**Authors: We have taken the control analysis suggestion from the reviewer and have implemented it in the manuscript to account for any effects of spatial proximity of the MTN for the path length analysis. Specifically, we identified a set of regions (outside of the MTN) with matched spatial proximity to the visual and default mode network/hippocampus and compared the path length between the visual and default mode network following removal of these proximity matched control regions to the path length following the removal of the actual MTN. We found that removal of the actual MTN resulted in a longer path length (i.e. greater disruption) compared to removal of the proximity matched control regions. This analysis can be found on page 9 and 10 and is shown for ease below.**

 However, another explanation for this effect is that the MTN is spatially the most proximal to both the visual network and DMN. Thus, we identified a set of 32 non-MTN regions that was matched for distance to both the visual and DMN compared to the MTN. Removal of the MTN nodes still resulted in a significantly higher pathlength between the DMN and visual network compared to removal of these spatially proximal matched control regions, t(39) = 5.5, p < .001. Further, removal of these control regions was not significantly different in terms of the resultant path length compared to the frontal parietal network, t(39) = .7, p = 0.5, though it was significantly higher than removal of the other networks (all t(39) > 3.2, all p < .001).

**And for the hippocampal spatial proximity analysis, we have added a section on page 11.**

To account for spatial proximity, as above, we identified a set of 31 non-MTN regions that was matched for mean Euclidean distance to the hippocampus and visual network relative to the MTN. Removal of these spatial control nodes was not sufficient to account for the effects of MTN removal, as removal of the MTN still led to significantly greater increase in path length, *t*(39) = 7, p < .001.

**We have also added a section in the methods on page 36 that explains how we performed this control procedure.**

We also ran analyses to control for spatial proximity. Path length results following removal of the MTN may be driven by the MTN’s spatial proximity to the visual network as well as the DMN/hippocampus. Thus, we identified a set of 32 non-MTN regions that was matched for distance to both the visual and DMN compared to the MTN. We also were able to identify 31 non-MTN regions that were matched for spatial distance to both the visual network and hippocampus compared to the MTN. To do so, we calculated the region-to-region Euclidean distance for each pair of ROIs for each subject. We then calculated the average distance between each MTN ROI to each DMN ROI, the average distance between each MTN ROI and each VIS ROI, and the average distance between each MTN ROI and hippocampal ROI, separately for each hemisphere. Then, we identified 2 sets of control ROIs, one such that the average distance between this control set and the DMN and the control set and the visual network would be within ±1 SD from the mean MTN-DMN and MTN-visual network distance and the other control set was the same except instead of controlling for MTN-DMN distance it controlled for MTN-hippocampal distance. Having found these sets that fulfilled these criteria, we deleted the sets and recalculated path length between the visual network and DMN and visual network and hippocampus and compared the resulting path lengths to deletion of every other network using paired t-tests.

Also, I would be curious to hear a bit more discussion re: why the MTN should mediate between the DMN and VN in the first place. For example, recent work has highlighted a relationship between the hippocampus/surrounding medial temporal lobes and the visual system (Shen et al., 2016; Ryan et al., 2020) - (how) does this finding extend this line of inquiry? Alternatively, the authors mention the idea that the MTN might represent "content" while regions of the DMN instead may represent "process" (or abstract, schema-like information). How would this fit with previous studies reporting pattern reinstatement of representations from encoding during recall, often specifically within DMN (in both AT and PM subnetworks, I believe) (e.g., Thakral, Wang & Rugg, 2017; Chen et al., 2017)?

**Authors: We thank the reviewer for guiding us to these studies and this literature. We have included mention of these papers in the discussion on page 21 and 22, noting the implicated role of medial temporal regions in both perception and memory-related eye movements. We have delved more deeply into discussions of reinstatement evidence from Chen and note that reinstatement occurred in both DMN and MTN regions in this paper. This reinstatement is a product of coarse patterns that are averaged over the time course of an event (often spanning 10s of seconds to minutes). Other research from the same group has shown evidence of abstracted representations which they referred to as event schemas in the DMN (Baldassano et al, 2018) and we have added in mention of this work as well on page 20 and page 21.**

Indeed, recent evidence has shown that regions in the MTN are more active during recall focussed on perceptual features (39), and during encoding of events in the absence of a strong semantic framework (40), whereas regions in the DMN are more active during recall focussed on conceptual or thematic elements (39), and during encoding of events that can be interpreted in the context of prior knowledge (40). Further, multivariate patterns in DMN regions can classify broad event types like restaurant vs. airport events, suggesting that they have represent abstract event structure (92).

Although these studies support a distinction between the MTN and DMN, it is important to note that these networks likely work in tandem to support retrieval of both content and structure, such as during recall of naturalistic events spanning minutes (93). Rather than viewing these systems as completely independent, a more measured interpretation is that task demands may determine what networks are recruited for retrieval.

(2)	How generalizable/reliable is the reported network parcellation/community-breakdown?

The authors parcellation of whole-brain networks generally replicates existing work. However, there were a few important differences. For example, both PRC and PHC were part of a common MTN network, rather than the originally proposed AT and PM systems (e.g., Ranganath & Ritchey, 2012). Also, earlier breakdowns of DMN connectivity described a core-DMN subsystem, consisting of the mPFC and PCC, which was not observed here (e.g., Andrews-Hanna et al., 2010).

As a reader, I am not sure how much weight I should be putting into interpreting these findings - despite their theoretical importance (e.g., for PMAT). For one, the present study was based on 45 subjects (though, 25-minute scanning), and the group-averaged functional connectivity matrix was submitted to the community detection algorithm. Considering this study was based on resting state, is there a reason not to replicate this parcellation in a separate sample of subjects (e.g., using a publicly available dataset) to confirm the generalizability of the reported network parcellation? Or, perhaps, calculating the split-half reliability of these network breakdown within the existing sample could be another possibility? Also, from what I understand, reliable resting state network estimation can be achieved with considerably shorter scans (e.g., Birn et al., 2013), so perhaps splitting subjects' scans in half and replicating the network parcellation could be another possibility.

In any case, I would like to hear the authors thoughts on the issue of interpreting the specific reported network breakdown, without a good sense of out-of-sample generalizability or within-sample reliability.

**We thank the reviewer for pushing us to further assess the generalizability of these results. As noted in our response to Reviewer 1 above, we addressed the concern about generalizability in both of the ways suggested by Reviewer 2. First, we performed a split-half analysis of the primary dataset finding very high replicability with ~95% of the regions being classified in the exact same network across split half replications. Importantly, the MTN reliably dissociated from the DMN in both groups. The methods and results for this have been added into the paper with a supplementary figure displaying the community labels on the cortical surface for both split half groups.**

**Second, we downloaded a publicly available dataset and re-ran the community detection. We again found high consistency and also observed a distinction between the MTN and DMN. Having replicated the distinction between the MTN and DMN with both split-half analyses and an external replication sample, we can be confident in the reliability of this finding.**

**The only difference between results from the two datasets was that, in the WashU dataset, parcels in the anterior temporal lobes separated into a module that exhibited very low functional connectivity with other regions. Analyses revealed that this deviation was because these regions had very low temporal signal to noise ratios (tSNR) in the secondary dataset. We think that this finding helps to demonstrate the added value of our dataset, which used a scanning protocol that was optimized for high tSNR in medial temporal lobe regions.**

**These results are described on page 6 and 7 in the revised manuscript and the relevant revisions are presented above in the response to Reviewer 1, question 1.**

**Reviewer 2 also mentioned that it was unclear how to interpret the present findings to previous models such as the PM/AT model proposed by Ranganath & Ritchey (2012). We have expanded on this issue in the discussion on page 18, including a figure (new labels have been added to the figure) to demonstrate the differences in partition solutions. In brief, we state that previous descriptions of the PM and AT networks were based on studies (e.g., Libby et al. 2012, *J Neurosci*; Kahn et al. 2008, *J Neurophysiology*) that focused on differences in functional connectivity with perirhinal and parahippocampal cortex (PRC and PHC) which necessarily puts them in separate networks, compared to the whole brain network approach used in the present study.**

**We note that our partition of the DMN is similar to previous data-driven such as that observed in Andrews-Hanna et al (2010), but explain that our partition differs in that the mPFC and posterior medial cortex dissociate in terms of network membership into their own separate networks. We also note that Andrews-Hanna et al (2010) manually defined a subnetwork that they referred to as the “core network” consisting of the mPFC and PCC based on the graph theory metric “betweenness centrality” (the mPFC and PCC both had high betweenness centrality which signifies that network communication often travels through these nodes). Thus, this core network was not defined explicitly by their partitioning algorithm.  This has been noted at the bottom of page 21.**

Andrews-Hanna et al (41) fractionated the DMN, identifying an MTL subnetwork that resembles our MTN, a dorsal medial subnetwork that resembles what we have termed the AT network, and a “core midline” network comprising both PCC and mPFC. This midline network differs from our partition which separates the mPFC and PCC, but it is important to note that the core midline network described in Andrews-Hanna et al. was created based on high betweenness centrality (a graph theory metric thought to be related to “hubness”) of the mPFC and PCC, rather than from a data-driven clustering procedure.

(3)	Why do regions within the same network have similar representational profiles?

I really appreciate the inclusion of this dataset. I think it adds quite a bit to the resting state results. However, the lack of detail regarding experimental procedures and what made up the RSA matrix made it hard to fully understand. The Methods section mentions that more details can be found in the Supplementary Information, but I didn't seem to find any there…(unless I somehow missed it?).

**We thank the reviewer for pointing out the mistaken omission of the supplemental details of this analysis. A more in-depth breakdown of the methods is now included in the supplementary information.**

Supplemental Methods

Pattern similarity analysis

Multivoxel pattern similarity analyses were performed on the fMRI data from the decision task runs. The analyses were conducted on unsmoothed functional images in the native space and the EPI timeseries underwent motion correction and high-pass filtering (0.01 Hz) in FMRIB’s Software Library (FSL; https://fsl.fmrib.ox.ac.uk/fsl/fslwiki).

For each trial, a single beta image was estimated by single trial models, based on the approach called Least Squares Single (LSS), for event-related blood oxygenation level-dependent (BOLD) signal change, controlling for signal change due to all other trials and motion artifact, using ordinary least squares regression, resulting in 128 single-trial beta images (1,2). Parameter estimates for each trial were computed using a general linear model, with the first regressor as a stick function placed at the onset of each trial and a second regressor containing all the other trials.

Single-trial beta images from run 2 were coregistered with single-trial beta images from run 1 using FSL’s FLIRT linear registration software (6 degrees of freedom). Coregistered single-trial beta images with atypically high mean absolute z-score (based on the distribution of beta estimates for each grey matter voxel across all trials) were excluded from further analysis. Based on a mean absolute z threshold of 1.5, between 0 and 10 trials were excluded per subject with a median of 4. Beta images went through a second visual inspection to make sure all the deviant trials were excluded. This noise trial exclusion procedure is adopted from the previous pattern similarity studies in the lab (3).

The representational similarity analyses were then conducted using the RSA toolbox by Nili et al. (4). For each region of interest, all trial patterns were correlated with each other using Pearson's r resulting in a 128 * 128 pattern similarity matrix for each ROI. The 128 * 128 pattern similarity matrix for each ROI was then filtered to exclude pairwise combinations that consisted of a trial with a bad quality beta. Then, the filtered 128 * 128 pattern similarity matrices for each ROI were vectorized and correlated for each pair of ROIs using Spearman’s correlation to assess the similarity of the representational profiles.

It would be helpful to get more details about the RDM used from the RSA analyses. I understand that there were 8 grocery items and 4 different grocery stores. So, was the RDM a 32 x 32 matrix, based on each combination of item and store? Or was it just a trial x trial similarity matrix? In any case, a schematic of the RDM in the Supplementary could be really helpful for readers in their attempts to parse this analysis and its conclusions.

Also, I'm quite curious as to why the authors observed more similar 'representation profiles' in ROIs within as opposed to across networks. Can we leverage the experimental paradigm to get a bit more insight into what psychological features drive this result?

For example, to go back to the MTN (point #1), the authors argue that the MTN may "provide [a] high-level feature representation of the external world" (p 18, ln 394). Can we find any evidence for that in the RSA analysis? For example, are representations more fine-grained in the VN than MTN than DMN? Couldn't this be quantified to some extent by looking at the off-diagonal variability in the ROI-level RSA matrices (more distinct patterns across trials, more fine-grained neural representations)? It's not perfect, as regions with noisier patterns of multivoxel activity might also produce this effect, but it seems in line with the authors' predictions.

Alternatively, if the RDM was based on objects (grocery items) and contexts (grocery stores), could the authors test whether context or item drives the similarity effects in these different networks? The authors' thoughts on what is actually driving the representational similarity would be appreciated and could benefit some predictions made in the manuscript.

**To first address why regions within the same network have similar network similarity profiles, we hold the assumption that if two regions are within the same network, they may have similar roles to play during cognition. To the extent that regions within the same network are processing shared information (e.g., about stimulus features or task demands), we might expect them to have similar representational profiles (i.e., voxel pattern information). Based on this reasoning, we predicted that second-order pattern similarity should be higher across regions within the same network than across regions in different networks. The intent of this analysis was to provide support for the idea that the network parcellation is relevant for understanding cognition. It is beyond the scope of this paper to provide a detailed treatment of this task fMRI dataset, but a separate manuscript describing behavioral results and hypothesis-driven analyses on that dataset is currently under revision at another journal (for preprint see Mizrak et al., 2019, BIORXIV). The key finding from our analyses of the Mizrak et al. data is that within network RDM similarity (which was entirely data-driven and blind to the particular theoretical questions that the task was designed to address) was higher than between-network similarity. These findings support the idea that the network parcellation provides useful information about representational content during cognitive tasks.**

Minor comments:

Page 6, Line 139: "mixed connectivity"

-  	What does "mixed connectivity" mean? Across-subjects? If so, perhaps a ratio of subjects that showed these effects would be helpful.

**When discussing “mixed connectivity” we meant that these networks had significant anterior, but not posterior functional connectivity and so the functional connectivity to the hippocampus was mixed; the use of the word “mixed” has been removed to avoid confusion.**

Page 7, Line 147: "higher-order feature encoding"

-  	I'm not sure what this means. Some additional elaboration could be helpful.

**We have changed the word “encoding” to “representation” and added a parenthetical to briefly clarify what we mean by higher-order feature representation on that line which is on page 8, now.**

A recent review discussing the heterogeneity within the PM network suggested that parahippocampal, and lateral parietal cortex interface with lower level sensory cortex in service of higher-order feature representation (i.e. perspective invariant representation of items and spaces) prior to binding in the hippocampus (29, 38, 56)

Page 10-11: Participation coefficients

-  	General question - what's the difference between these participation coefficient analyses and the network-holdout style analyses? In other words, does a high within-DMN participation coefficient imply that removal will lead to an overall increase in path length as compared to a network with lower within-DMN participation? In general, the participation coefficient analyses seemed to not have too much background (relative to the other analyses). Adding a sentence at the beginning of this section on why this matters could be helpful.

does a high within-DMN participation coefficient imply that removal will lead to an overall increase in path length as compared to a network with lower within-DMN participation?

**High within-DMN participation coefficient does not necessarily mean that removal will result in increased path length within the DMN.  Nodes with high participation have high diversity of connectivity to nodes in other networks. This can sometimes mean that removal of a high participation node will lead to an increase in path length, but if that particular high participation node is not representative of the shortest path between other networks, then its removal will not affect path length. We have added a few sentences at the beginning of the section on page 12 to further describe participation and the aim of the analysis.**

Nodes with a high participation coefficient are connected not only within their own community, but also communicate relatively more with nodes in other communities. This makes them well positioned to serve as regions that can integrate information from or coordinate activity towards multiple communities. Disruption of such nodes can lead to altered network function and cognitive impairment (62–65). Here, we examined the diversity of connections for the nodes in each of the DMN subnetworks to examine how information may be assimilated into the DMN from communities across the brain, and how information may be integrated within the DMN itself.

-  	"whereas nodes in the AT subnetwork and PM subnetwork may interface with networks outside of the DMN." Did the PM have a significantly higher participation coefficient than the MP when looking at extra-DMN connections? Was that comparison reported?

**This comparison was not reported and the language used previously “...and PM subnetwork may interface with networks outside of the DMN.”** **has been removed from the manuscript**

Figure 7

-  	Including the network labels here, on the figure itself, would be helpful.

**We have updated the figure to include this suggestion**

Figure 8

-  	The ROIs (on the left side) should have labels on the figure. It would make it more immediately legible.

**We have updated the figure to include this suggestion**

References:

Andrews-Hanna, J. R., Reidler, J. S., Sepulcre, J., Poulin, R., & Buckner, R. L. (2010). Functional-anatomic fractionation of the brain's default network. Neuron, 65(4), 550-562.

Birn, R. M., Molloy, E. K., Patriat, R., Parker, T., Meier, T. B., Kirk, G. R., ... & Prabhakaran, V. (2013). The effect of scan length on the reliability of resting-state fMRI connectivity estimates. Neuroimage, 83, 550-558.

Chen, J., Leong, Y. C., Honey, C. J., Yong, C. H., Norman, K. A., & Hasson, U. (2017). Shared memories reveal shared structure in neural activity across individuals. Nature neuroscience, 20(1), 115-125.

Ryan, J. D., Shen, K., & Liu, Z. X. (2020). The intersection between the oculomotor and hippocampal memory systems: empirical developments and clinical implications. Annals of the New York Academy of Sciences, 1464(1), 115.

Shen, K., Bezgin, G., Selvam, R., McIntosh, A. R., & Ryan, J. D. (2016). An anatomical interface between memory and oculomotor systems. Journal of Cognitive Neuroscience, 28(11), 1772-1783.

Thakral, P. P., Wang, T. H., & Rugg, M. D. (2017). Decoding the content of recollection within the core recollection network and beyond. Cortex, 91, 101-113.

Reviewer #3: In this paper, Barnett and colleagues provide an updated framework for understanding how resting state networks are organized, and how they interact with the hippocampus. This work takes a data-driven approach to investigate the well-studied "default mode network" or DMN, and illustrates that it can be 1) differentiated from a "medial temporal lobe network" (MTN) and 2) can itself be split up into three sub-networks. I reviewed this paper before for another journal and I was pleased to see how the authors have incorporated the previous reviewers' suggestions and updated the manuscript accordingly. I would like to commend the authors for making the connection between the current work and the previous PMAT framework much more clear. I have only a few minor comments.

1) 	The part of the paper that confused me the most, as a relative outsider to the graph theoretical world was the section on lines 217-245.  These results made slightly more sense to me after reading the discussion (lines 465-475), but perhaps some of these analyses could be better motivated up front so that the reader can better appreciate the importance of these results (and the interpretation of Figure 4).

**We thank the reviewer for this suggestion and have changed the manuscript on page 12 to better introduce the graph theory findings:**

Nodes with a high participation coefficient are connected not only within their own community, but also communicate relatively more with nodes in other communities. This makes them well positioned to serve as regions that can integrate information from or coordinate activity towards multiple communities. Disruption of such nodes can lead to altered network function and cognitive impairment (62–65). Here, we examined the diversity of connections for the nodes in each of the DMN subnetworks to examine how information may be assimilated into the DMN from communities across the brain, and how information may be integrated within the DMN itself.

2) 	Figure 6 depicts the representational similarity analysis results for the DMN sub-networks and the MTN.  I am curious how these similarity values change when you compare the similarity between the MTN and DMN seeds and the other resting state networks (e.g. Visual, language, etc). I am assuming that similarity would be lower, but are they significantly lower than the ones included/plotted?  For completeness this could be included in a supplemental figure or table.

**We have added in a supplemental figure showing the RDM similarity between the DMN subnetworks, and MTN to every network in the brain and we have referenced this in the manuscript original figure caption in the manuscript**

(see S6 Figure for the Representational Profile similarity of the cortico-hippocampal networks compared to every other cortical network).

**S6 Figure**

S6 Figure Caption

Boxplot of average region-to-region representational profile similarity between each cortical-hippocampal network (labelled as Target) to itself and every other network in the brain (labelled along the x-axis) across the sample. AUD, auditory; AT, anterior temporal; DAN1, dorsal attention network 1; DAN2, dorsal attention network 2; FP, frontoparietal; Lang, language; MP, medial prefrontal; MTN, medial temporal network; PM, posterior medial; SAL1, salience 1; SAL2, salience 2; SM, somatomotor; VIS, visual.

3) 	In the legend of Figure 2, there are color keys for the anterior and posterior hippocampus, but I don't see these colors in this particular figure.  Am I missing something or should those colors be removed?

**The hippocampi are in the force directed plot, they are just maybe less noticeable because there are only four nodes for them. Below I have circled them.**

4) 	On line 133, it is stated that: "The posterior hippocampus was defined as all the hippocampus posterior to the last slice of the uncal notch." (also in Methods on lines 632 and 633). I believe that the authors meant the "uncal apex" as described by Poppenk et al., (2013).

**That is correct. We have updated the manuscript with this correction.**
