# Supplementary material for: Intrinsic connectivity reveals functionally distinct cortico-hippocampal networks in the human brain
Source: PLoS Biol. 2021 Jun 2;19(6):e3001275. doi: 10.1371/journal.pbio.3001275 (PMC8202937; doi:10.1371/journal.pbio.3001275)
Supplement: Attachment — Submitted filename: Response to Reviewers 2.docx [file pbio.3001275.s012.docx]

Response to Reviewers

**Reviewer #1**: The authors have addressed my concerns. Nice job!

Response: Thank you

**Reviewer #2**: The authors have answered all of my questions and concerns. Their split-half reliability analysis, the external validation in a new sample and their control analysis for distance are important additions to the final manuscript (which I believe will make an important contribution to the field). I was not able to see the updated figures for this revision, as they did not seem to be attached.

Response: Thank you

**Reviewer #3**: I am happy with the authors' responses and I am looking forward to seeing the acceptance of the revised manuscript.

Response: Thank you
